# Supplementary material for: Comparison Study of Diagnosis and Treatment Planning for Dental Infections between Dental Students and Practitioners
Source: Healthcare (Basel). 2022 Jul 26;10(8):1393. doi: 10.3390/healthcare10081393 (PMC9330809; doi:10.3390/healthcare10081393)
Supplement: Supplementary file 1 [file healthcare-10-01393-s001.zip › healthcare-1824326-supplementary.pdf]

Case A

A 73-year-old female presented to the clinic, reporting that her gingiva around the maxillary right central incisor was swollen (Fig 1A; the black arrow).

(1) Medical History: BP = 130/75. No specific medical diagnosis was known. She was not taking any medication at that time.

(2) Dental History

The crown on the maxillary right central incisor was placed by another provider at a private practice. The patient said she had a PFM crown due to trauma. She lost most of the tooth structure at the age of 12. The tooth never had any problems until the gingival swelling occurred 3 weeks ago. There was no pain from the maxillary right central incisor reported by the patient.

(3) Clinical and Radiographical Evaluation

- Upon probing, pus was discharged from the mesiofacial site of the maxillary right central incisor with a 7-mm pocket depth.
- The probing depths from other sites from the maxillary right central incisor were 2 to 3 mm without bleeding on probing.
- No mobility was detected on the maxillary right central incisor.
- Interproximal crestal bone level was shown in Fig 1B.
- Negative response to percussion
- Mild response to palpation
- Lymphadenopathy: none
- Previous radiograph obtained in August 2014 presented in Fig 1C.

This patient was referred to an endodontist, who made the following evaluations. "The EPT test was impossible due to the crown. Cold test was not reliable. The cone beam computed tomography (CBCT) was obtained. Partially calcified pulp canals were noted in the CBCT (Fig 1D). Root fracture did not appear to be evident in the CBCT."

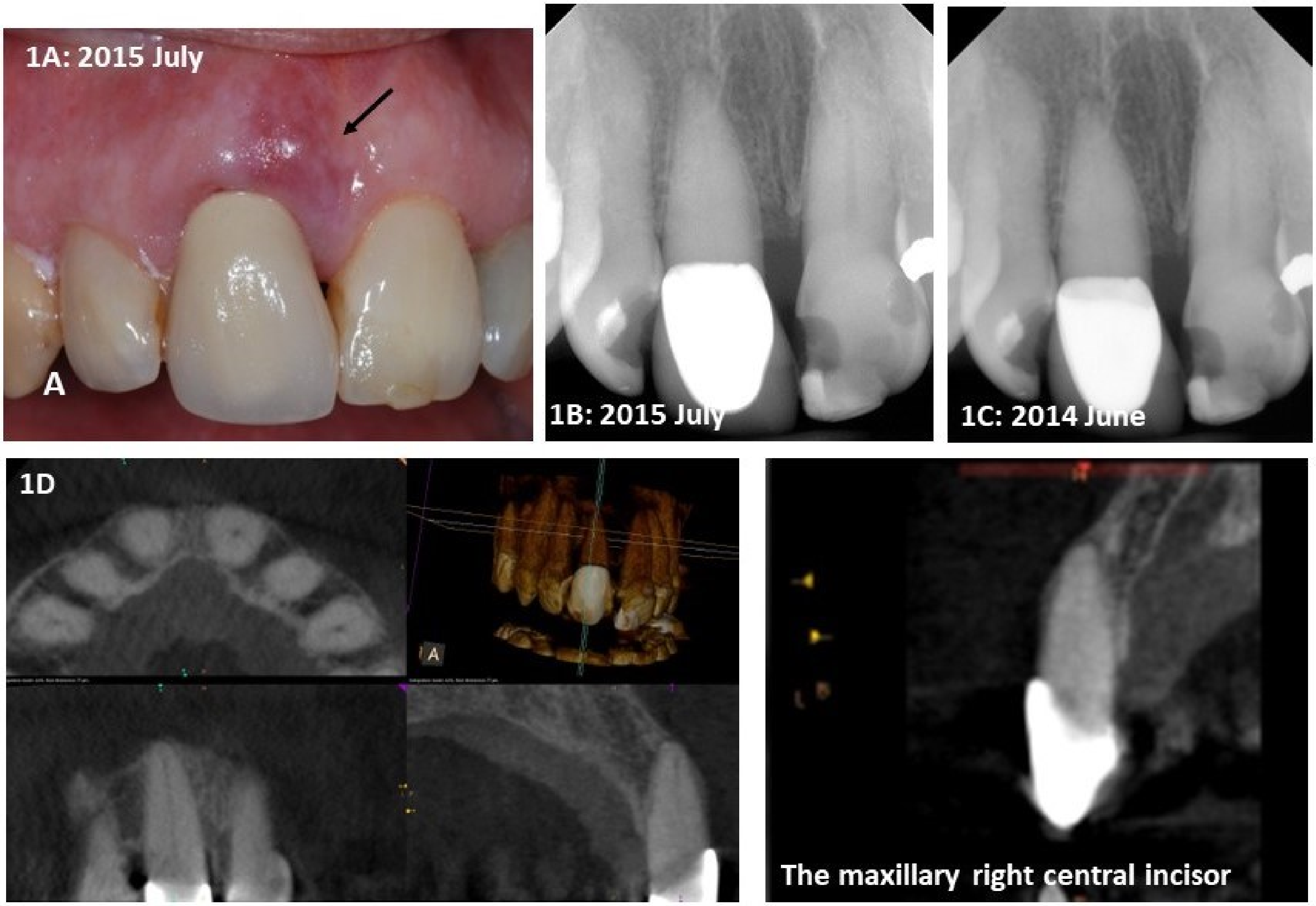

This patient came to you for a second opinion because a definitive diagnosis was not made even with the CBCT. You recommended an exploratory surgery for diagnostic purpose. After periodontal flap reflection, a lesion is visible in Fig 2A and 2B.

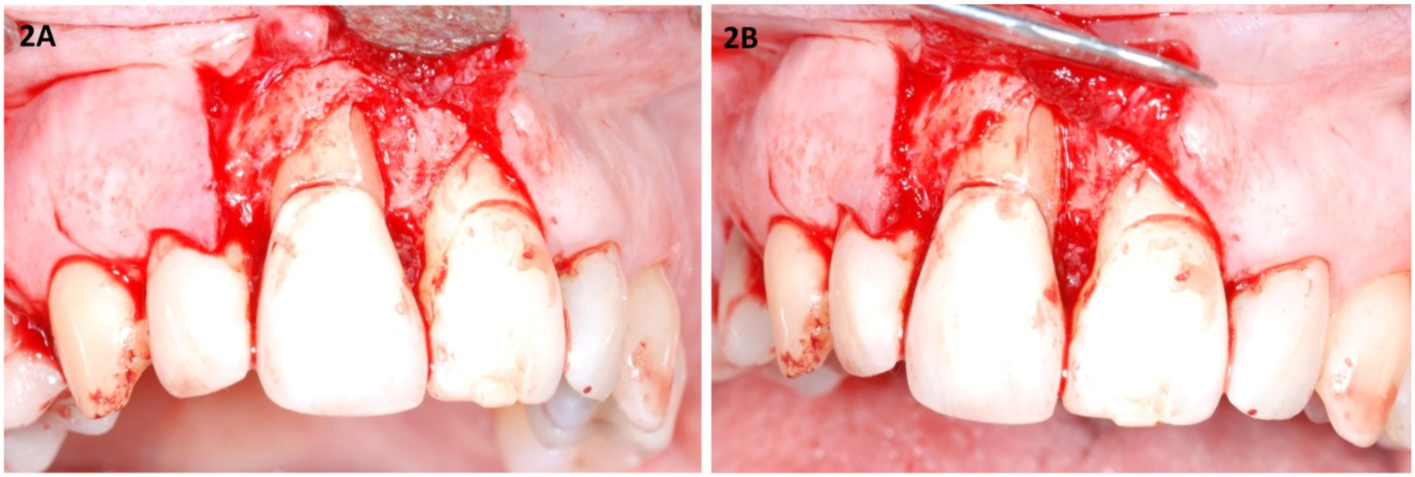

1. Based on the clinical and radiographical evaluation presented, what is the most appropriate diagnosis of the maxillary right central incisor?

- Lateral periodontal cyst
- Radicular cyst
- Incisive canal cyst
- Odontogenic keratocyst
- Apical periodontitis
- Necrotic pulp
- Gingivitis
- Periodontitis
- Pulpitis
- Periapical abscess
- Gingival abscess
- Periodontal abscess
- Pyogenic granuloma
- Occlusal trauma
- Endodontic-periodontal combined lesion
- Vertical root fracture
- Invasion of biologic width
- Other

2. Based on the clinical and radiographical evaluation, assign the prognosis for the maxillary right central incisor using McGuire and Nunn prognosis system (1996).

Good	Control of the etiologic factors and adequate periodontal support as measure clinically and radiographically to ensure the tooth would be relatively easy to maintain by the patient and clinician assuming proper maintenance.
Fair	Approximately 25% attachment loss as measure clinically and radiographically and/or Class I furcation involvement. The local and depth of the furcation would allow proper maintenance with good patient compliance.
Poor	50% attachment loss and Class II furcations. The location and depth of the furcation would allow proper maintenance, but with difficulty.

Questionable	>50% attachment loss resulting in a poor crown/root ratio. Poor root from. Class II furcation not easily accessible to maintenance care, or Class III furcations. ≥2+ mobility. Significant root proximity.
Hopeless	Inadequate attachment to maintain the tooth in health, comfort, and function. Extraction was performed or suggested.

3. What are your primary determinants for assigning the prognosis for the maxillary right central incisor?

- Pocket depths
- Tooth mobility
- Approximate % bone loss
- Signs and symptoms
- Root surface morphology
- Restorability
- Unknown pulpal diagnosis
- Periodontal diagnosis
- Other

4. In your opinion, what is the most appropriate treatment option for this patient's maxillary right central incisor?

- Extraction and immediate implant placement
- Extraction and bone graft procedure for future implant placement
- Extraction and a fixed bridge
- Enucleation of cyst and systemic antibiotics
- Root canal therapy
- Root end surgery
- Surgical debridement and systemic antibiotics
- Guided tissue regeneration and systemic antibiotics
- Other

Case B

A 65-year-old male presented for clinical evaluation of implant placement for the missing mandibular right first molar in January 2013.

1) Medical History: BP = 137/93. He has been diagnosed with hypertension 2 years ago and taking hydrochlorothiazide 20 mg/day.

2) The mandibular right first molar was extracted in June 2011; surgical ridge preservation utilizing bone graft materials was performed at the same.

During your clinical evaluation, you noticed a sinus tract. Patient did not feel any discomfort or pain. Root canal therapy (RCT) for the mandibular right second molar was performed 4 months ago in September 2012. Sinus track tracing with a gutta percha was performed. The limited periodontal evaluation was performed. Previous x-rays in 2012 are presented.

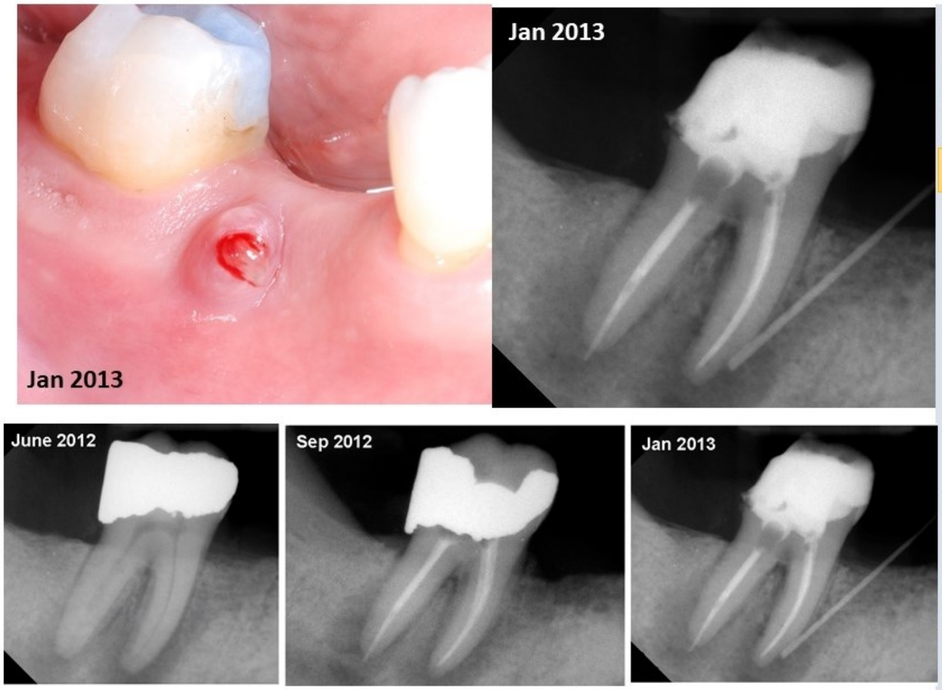

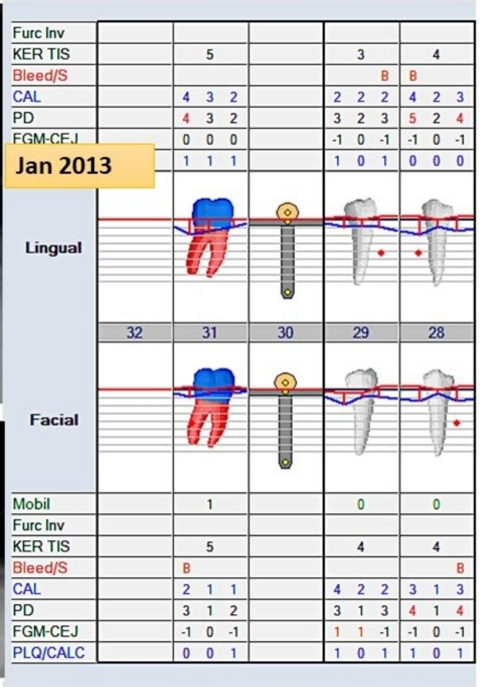

	32	31	30	29	28
Furc Inv					
KER TIS		5		3	4
Bleed/S				B	B
CAL		4 3 2		2 2 2	4 2 3
PD		4 3 2		3 2 3	5 2 4
FGM-CEJ		0 0 0		-1 0 -1	-1 0 -1
[illegible]		1 1 1		1 0 1	0 0 0

	32	31	30	29	28
Mobil		1		0	0
Furc Inv					
KER TIS		5		4	4
Bleed/S		B			B
CAL		2 1 1		4 2 2	3 1 3
PD		3 1 2		3 1 3	4 1 4
FGM-CEJ		-1 0 -1		1 1 -1	-1 0 -1
PLQ/CALC		0 0 1		1 0 1	1 0 1

5. Based on the clinical and radiographical evaluation presented, what is the most appropriate diagnosis of the mandibular right second molar?

- o Lateral periodontal cyst
- o Radicular cyst
- o Symptomatic apical periodontitis
- o Necrotic pulp
- o Gingivitis
- o Periodontitis
- o Pulpitis
- o Periapical abscess
- o Gingival abscess
- o Periodontal abscess
- o Occlusal trauma
- o Endodontic-periodontal combined lesion
- o Vertical root fracture
- o Invasion of biologic width
- o Other

6. What is the most appropriate prognosis for the mandibular right second molar based on the clinical and radiographic evaluation?

favorable	Comprehensive periodontal treatment and maintenance will stabilize the status of the tooth. Future loss of periodontal support is unlikely.
questionable	Local and/or systemic factors influencing the periodontal status of the tooth may or may not be controllable. If controlled, the periodontal status can be stabilized with comprehensive periodontal treatment. If not, future periodontal breakdown may occur.
unfavorable	Local and/or systemic factors influencing the periodontal status cannot be controlled. Comprehensive periodontal treatment and maintenance are unlikely to prevent future periodontal breakdown.
hopeless	The tooth must be extracted.

7. What are your primary determinants for determining the prognosis for the mandibular right second molar?
- Periodontal support
- Tooth mobility
- Root curvature
- Pulpal diagnosis
- Periapical diagnosis
- Restorability of the tooth
- Medical history
- Dental history
- The change in the size of periapical radiolucent lesion
- Reported success rate for treatment options
- Other

8. In your opinion, what is the most appropriate treatment option to be presented for the mandibular right second molar?
- Extraction and immediate implant placement
- Extraction and bone graft procedure for future implant placement
- Extraction and a removable partial denture
- Extraction and systemic antibiotics
- Enucleation of cyst
- Scaling and root planing
- Systemic antibiotics
- Root canal retreatment
- Root end surgery
- Re-implantation of the tooth (extraction of tooth, re-RCT, and re-implantation of the tooth)
- Surgical debridement
- Guided tissue regeneration
- Other
